# Levels of Tannins and Flavonoids in Medicinal Plants: Evaluating Bioprospecting Strategies

**DOI:** 10.1155/2012/434782

**Published:** 2011-09-29

**Authors:** Clarissa Fernanda de Queiroz Siqueira, Daniela Lyra Vasconcelos Cabral, Tadeu José da Silva Peixoto Sobrinho, Elba Lúcia Cavalcanti de Amorim, Joabe Gomes de Melo, Thiago Antônio de Sousa Araújo, Ulysses Paulino de Albuquerque

**Affiliations:** ^1^Natural Products Laboratory, Pharmacy Department, Federal University of Pernambuco, 50670-901 Recife, PE, Brazil; ^2^Laboratory of Applied Ethnobotany, Department of Biology, Federal Rural University of Pernambuco, Avenida Dom Manoel de Medeiros s/n, Dois Irmãos, 52171-900 Recife, PE, Brazil

## Abstract

There are several species of plants used by traditional communities in the Brazilian semiarid. An approach used in the search for natural substances that possess therapeutic value is ethnobotany or ethnopharmacology. Active substances that have phenolic groups in their structure have great pharmacological potential. To establish a quantitative relationship between the species popularly considered to be antimicrobial, antidiabetic, and antidiarrheal, the contents of tannins and flavonoids were determined. The plant selection was based on an ethnobotanical survey conducted in a community located in the municipality of Altinho, northeastern Brazil. For determination of tannin content was utilized the technique of radial diffusion, and for flavonoids, an assay based on the complexation of aluminum chloride. The group of plants with antimicrobial indications showed a higher content of tannins compared to the control groups. The results evidence suggests a possible relationship between these compounds and the observed activity.

## 1. Introduction

For centuries, extracts from plants have been used as folk remedies against various health problems [1], with many natural products leading to the development of clinically beneficial drugs [2]. According to Cox [3], the discovery of the medicinal benefits of these plants can be accomplished in two ways: by random selection or by selecting a target identified through phylogenetic (close relatives of plants known to contain useful compounds are included in the sample), ecological (plants in particular habitats with determinate life forms), or ethnopharmacological surveys (ethnodirected) (identifying plants traditionally used for specific diseases) [4].

The probability of finding useful compounds in plants by random selection is low (only 1 in 10,000 exhibits promising activities of interest to researchers), particularly in areas of high biodiversity such as the Caatinga, a typical semiarid ecosystem of northeastern of Brazil [5]. Already ethnobotanical surveys, according to Farnsworth quoted in [6], have contributed 74% of all medicines derived from plants. For example, in a study by Kaileh et al. [1] in a traditional Palestinian community, the selection of twenty-four folklore medicinal plants mentioned or described in the literature resulted in four potent cytotoxic species. In another study conducted in Mexico, the result was even more satisfying; antimicrobial activity was demonstrated in 75% of the plants that were tested, mainly for diseases of bacterial origin in the studied area [7]. 

Consequently, the ethnodirected approach has received significant attention in recent years [1]; several studies have been aimed at contributing to the development of this tool in the search for natural substances with therapeutic action in the northeast region of Brazil [8–11]. In that region, traditional knowledge about medicinal plants has long been an integral part of popular medical practice, being used by 90% of the economically disadvantaged population to treat their health problems [12].

The Caatinga is crucial to human life because it offers a wide variety of plant and animal resources for the survival of those who live there [8, 9, 13–16]. Among its benefits are the medicinal plants found within that are the basis of Brazilian popular medicine [10].

Many of the plant species used in Caatinga as folk medicines include phenolic compounds in their compositions, leading to the popular belief that the attributed therapeutic activities are associated with the presence of these compounds [8, 17–19]. Compounds with phenolic group in its structure have a pharmacological potential [20], which is represented by activities such as antimicrobial [21–23], hypoglycemic and/or antidiabetic [24–29], and antidiarrhoeal [30–33].

Previous studies have found phenols in 100% of a group of medicinal plants in the Caatinga [17, 19]. Moreover, in some species that have high value for local communities, the compounds of interest appear in high concentrations, often supporting therapeutic indications that are locally assigned [8].

Thus, our current research is focused on the following question: Is the content of phenolic compounds (tannins and flavonoids) in the groups of plants popularly indicated for diseases related to the activity of these compounds? We hypothesized that there would be a strong relationship between high levels of these compounds and the native species from Caatinga selected by an ethnobotanical survey. If this tendency was confirmed, it would then be possible to predict or direct studies of bioprospecting for medicinal plants of the region.

## 2. Material and Methods

### 2.1. Data Collection

Our study is based on an ethnobotanical survey conducted by the Laboratory of Applied Ethnobotany (LEA) from UFRPE that resulted in the creation of a database with information about the plant species used by the community. Their study was conducted in the rural area around Altinho, which is located in the central Agreste at 163.1 km from Recife (capital of Pernambuco State). The region has a total area 454.486 km² and a semiarid, hot climate [34]. In this study, after legal procedures such as collecting signatures of a Free and Informed Term of Consent from all persons over eighteen years of age in the community (see [35]), the ethnobotanical information was collected and divided into three stages: first, a general survey regarding the use and knowledge of medicinal plants with the community; second, local experts were selected based on the quality and quantity of information they provided in the first stage; third, the specialists were subjected to the technique of a free list, which consists of gathering information from a specific domain of knowledge [8, 19, 36–39].

### 2.2. Selection of Plants for the Study

To select plants for the study, a literature search was performed on the pharmacological activities that are common to the categories of secondary metabolites we had chosen to focus on: tannins and flavonoids. Various activities of phenolic compounds were identified: antimicrobial [21–23], antioxidant, anti-inflammatory, antidiarrhoeal [30–33], cardioprotective, and hypoglycemic and/or antidiabetic [24–29].

Subsequently, we identified which of these common activities were present as therapeutic agents in the medicinal plants surveyed in the Carão Community from the ethnobotanical survey mentioned above. The database from the overall ethnobotanical survey was filtered, with inclusion criteria that all species should be native (when they are native to South America) [9] in the Caatinga and that they possessed the selected therapeutic activities, to give a total of 25 plants (Table 1). In this community, 12 species were cited to have antimicrobial activity, but only nine were native. Similarly, 20 possessed antidiarrhoeic activity, but only 10 met the criteria. Finally, of the nine species that had antidiabetic and/or hypoglycemic action, only six were selected following the same reasoning (see Table 1). 

After the random choice of species (30), four groups were established to analyze the content of phenolic compounds.

- *Group 1*: random selection of 10 cited plants, based on the general survey, which served as the control group.
- *Group 2*: selection of nine plants with described antimicrobial indication based on the general survey.
- *Group 3*: selection of 10 plants with described antidiarrheal indication based on the general survey.
- *Group 4*: selection of six plants with described hypoglycemic and/or antidiabetic indication, based on the general survey.

### 2.3. Determination of Phenolic Compound Contents 

#### 2.3.1. Sample Preparation

Plant samples were collected according to the general ethnobotanical survey conducted by the LEA group from the specific parts of plants used by the community for each therapeutic indication. Each sample contained at least three individual plants of the same species that were mixed to compose a single sample. Samples were collected in June 2009. After drying at room temperature, the samples were crushed in an industrial crusher and standardized with an electromagnetic stirrer and sifters to obtain a granulometry of 60 Mesh. 

#### 2.3.2. Determination of Tannin Contents

The tannins were quantified by Hagerman's radial diffusion method [40] as adapted by Cabral et al. [41]. Thus, for the preparation of the gel was used a solution of 50 mM acetic acid and 60 mM ascorbic acid, adjusting the pH to 5 with the addition of sodium acetate, which was added in agarose (type I) (Sigma-Aldrich) 1%. Subsequently, the mixture was brought to the heating, stirring, until boiling point, so that there was a complete homogenization of agarose. After cooling (±45°C) was added to bovine serum albumin (BSA) fraction V fatty acid free (Sigma-Aldrich) at a concentration of 0.1%. Aliquots of 10 mL were distributed in Petri dishes with 9 cm in diameter that were placed on a level surface to which they were formed uniform layers of the gel. After total solidification of the gel, wells were made with a capacity of about 8 *μ*L, 2 cm distant from each other and the edges of the plates with a punch of 4 mm in diameter. The samples were analyzed in triplicate.

Samples (100 mg powdered) were extracted in 1 mL methanol: water 50% (v/v) for one hour at room temperature. Three successive aliquots of 8 *μ*L were applied to extract formed with the aid of micropipettes, directly in the wells. After complete absorption by the gel of aliquots of the extracts, the plates were sealed with parafilm and incubated at 30°C for 120 h. The extract containing tannin after reaction with albumin produces an opaque precipitate in the form of disc, from which the diameter squared is proportional to the concentration of tannins in the extract [40]. To construct the standard curve were used five different aliquots of a solution of tannic acid 25 mg/mL. We used Corel Draw X3 Version 13 to measure the diameters of the rings formed.

#### 2.3.3. Determination of Total Flavonoid Contents

Quantification of flavonoids was based on a procedure described by Amorim et al. [42] consisting of a spectrophotometric test at 420 nm, which was considered precise, reproducible, highly accessible, and highly practical. 

Samples of 500 mg of each plant were transferred to a 50 mL Erlenmeyer flask. Next, 25 mL of methanol pure for analysis (P.A.) was added, and the mixture was then heated on a hot plate at 80°C ± 5°C for thirty minutes. Finally, the extract was filtered through filter paper and transferred to a 50 mL volumetric flask.

The precipitate was washed with 25 mL of methanol and filtered again into the same flask, completing the volume of methanol. From this solution, 0.5 mL were transferred to a 25 mL volumetric flask. Into these flasks were added 0.6 mL of glacial acetic acid, 10 mL of a pyridine-methanol solution (2 : 8) and 2.5 mL of a 5% methanolic solution of aluminum chloride. Distilled water was then added to fill the flask. After 30 minutes at rest at room temperature, absorbance readings were taken by spectrophotometry at 420 nm. The samples were analyzed in triplicate.

For the standard preparation, 6.0 mL of methanol were added to a 10 mL volumetric flask followed by 5.0 mg of Rutin, purchased from Sigma-Aldrich. To achieve complete dissolution of the standard, the solution was maintained for five minutes in an ultrasound bath. Additional methanol was then added to complete the final volume of the flask. From this solution, aliquots of 0.1, 0.25, 0.5, 1.0, 2.5, and 3.75 *μ*g/mL were taken and transferred to 25 mL flasks.

To each flask were then added 0.6 mL of glacial acetic acid, 10.0 mL of pyridine 20%, and 2.5 mL of methanolic solution of aluminum chloride at 5%, followed by water to complete the volume. After resting for 30 minutes at room temperature, the absorbances were read at 420 nm in glass cuvettes.

A blank solution was prepared in a 25 mL volumetric flask, using all reagents described before (except the extract or standard), and its absorbance readings at 420 nm were taken as a white solution to zero the equipment.

### 2.4. Statistical Analysis of Data

The levels of tannins and flavonoids of each plant group were statistically compared to each other by the Kruskal-Wallis test. The relative proportions of these compounds between plants of the same group were determined using the *G* test from Williams based on Araújo et al. [8]. For the purposes of this test, tannin levels are typically considered high when >10% and low when <10%, and flavonoids high when >1% and low when <1%. However, we realized that for radial diffusion the percentage of tannins decreases by almost half [41]. Therefore, for our analysis, we considered that tannin levels were high when >5% and low when <5%. In all analyses, a power decision of *α* < 0.05 was assumed. All tests were performed using Software BioEstat 4.0 [43].

## 3. Results and Discussion

The average comparison in the tannin content was not significant in either group of therapeutic indications when compared to the control group. The group of species popularly thought of as antimicrobial ($\overset{̅}{x}=3.74\pm 4.78$) and antidiarrheal ($\overset{̅}{x}=2\pm 2.37$) had higher average tannin contents than the group of plants that were chosen at random ($\overset{̅}{x}=1.09\pm 1.79$). 

Studies have shown that tannins possess antidiarrhoeal [23, 32, 33, 44–49] and antimicrobial activities [50, 51], and, although the difference in tannin levels of these two groups was not significant in our study, compared to the random group of plants (Table 1), these compounds are more concentrated in plants with these activities [8, 17–19].

The group of antimicrobial plants exhibited proportionally greater occurrences of high levels of tannin compound when compared to the control group (*P* < 0.0001, *G* = 29.77), to the group of antidiarrheal plants (*P* < 0.0004, *G* = 12.75), or the group of antidiabetic plants (*P* < 0.0001; *G* = 68.33). Additionally, the antimicrobial group had the highest average tannin levels of the four analyzed, despite the fact that five of the nine species of this group did not register high levels. However, Alencar et al. [37] identified tannin in four of these five species: *Amburana cearensis* (Allemão) AC Smith, *Ziziphus joazeiro* Mart, *Erythrina velutina* Willd., and *Maytenus rigida* Mart. This discrepancy may have occurred because different methodologies were used.

*Mimosa tenuiflora* (Willd.) Poir., popularly known as jurema-preta, exhibited the highest tannin content (12.58) (Table 1). In the Caatinga community, it is used for its antimicrobial property that has been demonstrated pharmacologically [52–54]. The tannic compounds are believed to be responsible for most of this activity [54]. 

In a separate but related study based in the same region, Araújo et al. [8] identified *Anadenanthera colubrina* (Vell.) Brenan and *Myracrodruon urundeuva* Allemão, and not *Mimosa tenuiflora*, as the species possessing the highest levels of tannins. In their analysis, the quantification of tannins was performed by chemical precipitation of casein method to identify whether plants indicated as anti-inflammatory and/or healing were associated with the presence of these compounds. The discrepancy in determining which species exhibit higher levels of tannin may be rationalized based on the different chemical properties that were tested for using the two tannin-detection methods [41]. 

The plants listed popularly as antimicrobial, almost 50% of them, have a higher occurrence of tannins towards the group of random plants, elucidates the idea that groups of specific therapeutic indications can serve as a criterion to find Caatinga species with high levels of this compounds. This observation was verified by Araújo et al. [8] facing the species as inflammatory and/or wound healing in this region.

Regarding the presence of flavonoids, in all the species common to both our analysis and that of Alencar et al. [37], we observed a 100% agreement. However, just as in the case of the tannins, no significant differences between the averages of the groups with therapeutic indications were observed for the flavonoids (Table 2). The averages of antidiabetic plant groups ($\overset{̅}{x}=1.32\pm 1.22$) and antidiarrheal ($\overset{̅}{x}=0.94\pm 0.94$) were slightly higher than the control group ($\overset{̅}{x}=0.849+0.91$). 

None of the therapeutic indication groups displayed proportionally higher occurrences of flavonoids when compared to the randomized group. The group of antidiarrheal plants had the same proportion of species with high levels of flavonoids as the control group, and both showed a higher occurrence of these compounds compared with the antimicrobial plants (*P* < 0.0001; *G* = 21.33).

The diabetes group of plants also exhibited a higher occurrence of flavonoids when compared with antimicrobial plants (*P* = 0.0002; *G* = 13.42), indicating that in our study the flavonoids did not display a strong association with activity despite literature precedent showed the contrary [55–57]. Pharmacological evidence already exists of antidiabetic [56, 58] and antidiarrhoeal activities [58–60] derived from the presence of flavonoids in plant species.

Contrary to our expectations, due to the small sample size of the antidiabetic plant group, these species showed the highest average levels of flavonoids in relation to the other studied indications. Additionally, the species that had highest content of these compounds, *Bauhinia cheilantha* (Bong.) Steud (4.94) (Table 1), is popularly associated with this therapeutic indication. Da Silva et al. [61], in a study on the chemical compositions and pharmacological potentials of plants of the genus *Bauhinia*, mention that this activity has been scientifically proven in this species by the treatment of rats with diabetes induced by alloxan with its methanolic extract (600 mg/kg).

Thus, the fact that the group of antimicrobial plants possesses a higher proportion of plants with high tannin levels compared to the other therapeutic indications studied may serve as evidence for future analyses aimed at finding species with high levels of this compound that appear to be locally associated with this activity. However, we are aware that other compounds, not analyzed in this study, may exert a therapeutic action cited by the local community.

## Figures and Tables

**Table 1:** Medicinal plants analyzed with their levels of tannins (T) and flavonoids (F) in dry samples in an ethnobotanical survey conducted in the Caatinga vegetation in Pernambuco state, Northeast Brazil.

Group: indication	Scientific name	Popular name	Part used	*F* (%)	*T* (%)
Group I:	*Schinopsis brasiliensis *Engl.	Baraúna	Bark	2.55	5.53
random selection	*Hymenaea courbaril* L.	Jatobá	Bark	0.46	2.35
	*Handroanthus impetiginosus *(Mart. ex DC.)* Mattos *(*Tabebuia impetiginosa *(Mart. ex DC.) Standl.)	Pau d'arco roxo	Bark	0.1	—
	*Cereus jamacaru *P. DC.	Mandacaru	Cladode	0.2	—
	*Capparis jacobinae Moric. *ex Eichlera.	Incó	Leaf	1.29	—
	*Serjania lethalis *A. St.-Hil.	Ariú	Root	0.05	1.21
	*Manihot glaziovii * Muell. Arg.	Maniçoba	Bark	0.3	1.89
	*Nicotiana glauca *Graham.	Pára-raio	Leaf	1.6	—
	*Solanum aculeatissimum *Jacq.	Gogóia	Root	1.91	—
	* Crataeva tapia *L.	Trapiá	Bark	0.03	—

Group II:	*Amburana cearensis *(Allemão) A.C. Sm.	Imburana açu	Bark	0.33	—
antimicrobial	*Ziziphus joazeiro *Mart.	Juazeiro	Bark	0.14	—
	*Anadenanthera colubrina *(Vell.) Brenan	Angico/preto	Bark	0.39	8.24
	*Erythrina velutina *Willd.	Mulungu	Bark	0.21	—
	*Maytenus rigida *Mart.	Bom nome	Bark	0.3	—
	*Mimosa tenuiflora *(Willd.) Poir.	Jurema lisa	Bark	0.2	12.58
	*Caesalpinia pyramidalis *Tul.	Catingueira rasteira	Bark	0.65	6.01
	*Myracrodruon urundeuva *Allemão.	Aroeira	Bark	2.95	6.88
	*Guapira laxa *(*Netto*) Furlan.	Piranha	Bark	0.04	—

Group III:	*Libidibia ferrea *(Mart. ex Tul.) L. P. Queiroz (*Caesalpinia ferrea *Mart.)	Jucá	Bark	0.49	6.24
antidiarrheal	*Caesalpinia pyramidalis *Tul.	Catingueira rasteira	Bark	0.65	6.01
	*Lantana camara *L.	Chumbinho	Leaf	3.72	—
	*Croton blanchetianus *Baill.	Marmeleiro	Bark	0.47	2.47
	*Croton rhamnifolius *Willd.	Velame	Leaf	2.71	—
	*Eugenia uvalha *Cambess.	Ubaia	Bark	0.72	1.68
	*Spondias tuberosa *Arruda.	Umbu	Bark	2.26	1.51
	*Croton argyroglossum *Baill.	Rama branca/Velame branco	Bark	0.23	—
	*Ziziphus joazeiro* Mart.	Juazeiro	Leaf	1.75	—
	*Cedrela odorata* L.	Cedro	Bark	0.21	2.09

Group IV:	*Bauhinia cheilantha *(Bong.) Steud.	Mororó branco	Leaf	4.94	1.82
hypoglycemia and/or antidiabetic	*Croton argyroglossum *Baill.	Rama branca/Velame branco	Bark	0.23	—
	*Spondias tuberosa *Arruda.	Umbu	Bark	2.26	1.51
	*Tillandsia usneoides *(L.) L.	Salambaia comprida/Samambaia	Whole plant	0.3	—
	*Erythrina velutina *Willd.	Mulungu	Bark	0.21	—
	* Maranta divaricata *Roscoe.	Cana de macaco	Bark	0.02	—

—: not detected.

**Table 2:** Comparison between the amounts (%) of tannins and flavonoids in a herbal ethnodirected strategy selected from the vegetation of the Caatinga in the state of Pernambuco, northeast Brazil.

Group: indication	Tannins (average ± standard error)	Flavonoids (average + standard error)	Proportions of species with high versus low tannin content	Proportions of species with high versus low flavonoid content
Random selection	1.09 ± 1.79^a^	0.849 ± 0.91^a^	10.00/90.00^a^	40.00/60.00^a^
Antimicrobial	3.74 ± 4.78^a^	0.28 ± 0.17^a^	44.44/55.56^b^	11.11/88.89^b^
Antidiarrheal	2.0 ± 2.37^a^	0.94 ± 0.94^a^	20.00/80.00^a^	40.00/60.00^a^
Hypoglycemia and/or antidiabetic	0.55 ± 0.86^a^	1.32 ±1.95^a^	0.00/100.00^c^	33.33/66.67^a^

Averages or proportions followed by the same letter in column do not differ at 5% probability of Kruskal-Wallis.
